# The expression of stromal biomarkers in small papillary thyroid carcinomas

**DOI:** 10.1186/s12957-022-02805-w

**Published:** 2022-10-14

**Authors:** Haytham Bayadsi, George Barghout, Moa Gustafsson, Malin Sund, Joakim Hennings

**Affiliations:** 1 Department of Surgical and Perioperative Sciences/Surgery, Umeå University, Umeå, Sweden; 2 Department of Clinical Pathology, Sunderby Hospital, Sunderbyn, Sweden; 3 Clinicum/Department of Surgery, University of Helsinki and Helsinki University Hospital, Helsinki, Finland

**Keywords:** Papillary thyroid cancer, Tumor stroma, Type 1 collagen, Type IV collagen, Alpha-smooth muscle actin, Matrix metallopeptidase 9

## Abstract

**Background:**

The importance of stroma for tumor progression is recognized for many cancer types. In this study, we aim to evaluate the expression of types I (Col1) and IV (Col4) collagens, alpha-smooth muscle actin (a-SMA), and matrix metallopeptidase 9 (MMP-9) in the tumor stroma of small papillary thyroid carcinoma (PTC).

**Material and methods:**

Twenty-five non-metastatic small PTCs (pT1N0) and nineteen metastatic small PTCs (pT1N1b) including corresponding metastatic lateral lymph nodes were selected and paraffinized tissue blocks retrieved. The samples were stained for Col1, COL4, a-SMA, and MMP-9 antibodies using immunohistochemistry. The expression of the stromal proteins was scored and analyzed based on the location, intensity, and distribution.

**Results:**

Col1 and Col4 expression were significantly higher in normal thyroid tissue compared to PTC tissue. On the contrary, expression of a-SMA and MMP-9 was higher in PTC tissue compared to normal thyroid tissue. Both Col1 and Col4 were significantly more highly expressed in the non-metastatic tumors compared with metastatic tumors. The expression of a-SMA and MMP9 was slightly, but not significantly, higher in the metastasized tumors and their respective lymph nodes. There was a significant correlation between the metastasized tumors and their respective lymph nodes in Col1 and MMP-9 expression.

**Conclusions:**

Col1, Col4, a-SMA, and MMP-9 expression in PTCs differs significantly from that of normal thyroid tissue. The higher expression of Col1 and Col4 in normal thyroid tissue and in the non-metastasized tumors indicates that Col1 and 4 might have a potential protective role in tumor progression. The higher expression of a-SMA and MMP9 in PTCs indicates that these proteins might have a role in promoting PTC progression and aggressiveness.

**Supplementary Information:**

The online version contains supplementary material available at 10.1186/s12957-022-02805-w.

## Introduction

Thyroid cancer is the most common type of cancer in the endocrine glands [1, 2]. Its incidence is steadily increasing, with a current rate in Sweden of 550–600 new cases per year [3]. One etiologic factor is ionizing radiation, but this alone cannot explain the increasing incidence [3, 4]. One contributing factor is believed to be the widespread use of ultrasound examinations of the neck and fine-needle aspiration (FNA) of lesions found. FNA leads to increased diagnosis, but also allows for diagnosing patients at an early stage [5–8]. Small papillary thyroid cancers (PTCs) (≤ 20 mm in size) and especially papillary thyroid microcarcinomas (PTMC ≤ 10 mm) are responsible for most of the incidence rate increment [3, 8, 9].

PTC constitutes the most common subtype of thyroid cancer and generally has a good prognosis, with 5-year disease-free survival (DFS) at 87.5%, and cancer-specific mortality after 5 years at 0.8%. For differentiated thyroid carcinoma, the 10-year survival rate ranges between 85 and 95% [1, 2, 10].

Small PTCs are classified as extremely low risk or low risk, and both have a favorable prognosis [6, 7, 11, 12]. Yet some of these small PTCs can cause lateral lymph node metastases, distant metastases (most commonly in the lungs), or even death [1, 3, 7, 11]. These findings indicate that there is a difference in biological aggressiveness within this group. Known histological features that are associated with a worse prognosis are multifocality, bilateral foci, vascular invasion, extrathyroidal extension, and/or lymph node metastases [6, 7].

Earlier studies have shown that there is a connection between aggressiveness and mutations in the oncogenic BRAF V600E and loss of PTEN. KRAS, NRAS, and RET/PTC have also been studied [13–15]. Mutation in BRAF V600E and loss of PTEN have been shown to recruit fibroblasts to the cancer microenvironment. Additionally, this mutation commonly results in a constitutive activation of BRAF kinase and a constant stimulation of the mitogen-activated protein kinase pathway (MAPK) [13, 14]. Mutations in RET/PTC and RAS genes also activate cascades involving MAPK [14, 15]. It has been suggested that these different genetic alterations contribute to the progression of thyroid cancer [13, 14] with the presence of BRAF mutations predicting faster growth rate, spread, and higher cancer mortality [16].

The extracellular matrix (ECM) and the non-malignant cells of the tumor, such as immune cells, cells of the vasculature, and fibroblasts, are defined as the “tumor stroma” [17–19]. Fibroblasts in particular have been shown to play an important role in the stromal modulation and homeostasis of the ECM [20, 21]. The importance of stroma for tumor progression is recognized for many cancer types, including breast and pancreatic cancer, and findings indicate that the stroma could be a source of tumor markers. The stroma can also be used to predict aggressiveness and invasiveness and hence could be an important tool when planning each patient’s treatment [18, 19, 22–24].

Collagens constitute the most abundant protein type of the ECM, and type I collagen (Col1) is the most common collagen type in the human body. It forms fibrils and is an important structural part of connective tissues. Earlier breast cancer studies have implied that Col1 may play a part in metastasis, where a higher density of collagen was found in metastatic lymph nodes in mice with breast cancer than in non-metastatic lymph nodes [25]. Another study indicates that Col1 is an important component in the mediation of a malignant phenotype in pancreatic cancer through its effects on cell adhesion, migration, and proliferation [26].

In normal tissues, type IV collagen (Col4) is found exclusively in basement membranes (BM), which divide the epithelium from the stroma and add support to the tissue [27, 28]. Col4 forms a network in the basement membrane that influences cell adhesion, migration, and differentiation of epithelial cells. There have also been studies showing that Col4 fragments can act as anti-angiogenetic as well as pro-apoptotic substances, thus inhibiting tumor growth in vivo [23, 27–29]. Fibroblasts have been shown to contribute to these functions in the BM, partially by secreting Col4 [28, 30]. These findings suggest that Col4 can impact tumor progression and aggressiveness [31–33].

Alpha-smooth muscle actin (a-SMA, ACTA2) is an actin protein that is involved in the contraction apparatus for smooth muscle. a-SMA is found in many different cells with smooth muscle or myogenic differentiation including the vessels, muscularis mucosae, and propria and most importantly myoepithelial cells that can be used as markers for invasion [34, 35]. In normal wound healing, injury to the basement membrane results in an induction of fibroblast migration, proliferation, and differentiation. This leads them to become activated fibroblasts, so-called myofibroblasts, due to their expression of a-SMA [21, 34]. Stromal myofibroblasts are also found in primary and metastatic carcinomas such as in the prostate and pancreas, where it is believed that they play a central role in cancer cell growth, proliferation, stromal activation, and metastatic conversion [35–37].

Matrix metalloproteinase-9 (MMP-9, also known as 92-kDa gelatinase B type IV collagenase) is an important member of the MMP family, zinc-dependent extracellular proteases that cleave many ECM proteins to regulate the ECM remodeling and tissue architecture [38, 39]. MMP-9 plays a crucial role in many biological processes, such as wound healing and tissue repair. It plays a role in basement membrane degradation, which is an essential step in tumor invasion and metastasis. MMP-9 also facilitates the release of tissue‑bound fibroblast growth factor (FGF) and vascular endothelial growth factor (VEGF), thus contributing to tumor growth [38]. Many studies have shown that MMP-9 plays a role in promoting cancer development and progression through influencing the tumor microenvironment. MMP-9 has been found to be a potential cancer biomarker in several cancer types, such as non-small cell lung cancer, cervical cancer, pancreatic cancer, and metastasized breast cancers [38]. Previous studies have revealed higher levels of MMP-9 in more invasive thyroid cancer tissues, and a recent study even suggested that MMP-9 may be one of the factors influencing the high aggressiveness and poor prognosis for thyroid cancer due to the promotion of the epithelial-mesenchymal transition [40, 41].

Our aim was to study the expression of a set of different tumor biomarkers in normal thyroid tissue compared with PTC, and between the different tumor subgroups, i.e., non-metastatic (N0T) vs. metastatic (N1T). A second aim was to explore whether there is a correlation between the metastatic tumors (N1T) and their corresponding lateral lymph node metastasis (N1N) in terms of stromal protein expression.

## Materials and methods

### Collection of material

The inclusion criteria were histologically verified T1N0 and T1N1b tumors according to the AJCC/UICC pTNM classification, 8th Edition, 2017 [1, 42]. Twenty-five small PTCs without metastasis (pT1N0) and 19 (pT1N1b) with metastasis were selected from the Swedish National Quality Register for Thyroid Cancer. Tissues from primary tumors and lymph node metastases were then collected from three local biobanks as paraffin-embedded, formalin-fixed tissue samples. One of the patients in the metastatic group was excluded since the tissue samples were not available, and another patient in this group was excluded because the tissue sample was of poor quality. After these exclusions, the resulting final cohort consisted of 44 patients. Complementary information was collected from the patients’ medical and pathology records, such as tumor size, tumor side, nodal status, age at diagnosis, and method of surgery.

### Immunohistochemistry staining

Five-micrometer thick, paraffin-embedded tissue slices were cut and stained using four different antibodies: anti-Col1 antibody (rabbit polyclonal ab34710, Abcam, Cambridge, UK), anti-Col4 antibody (mouse monoclonal antibody, BM4067, OriGene, Maryland, USA), anti-aSMA antibody (rabbit polyclonal ab5694, Abcam, Cambridge, UK), and anti-MMP9 antibody (rabbit polyclonal ab38898, Abcam, Cambridge, UK). The antibodies were diluted with PBS as follows: anti-collagen I 1:800, anti-collagen IV 1:50, anti-aSMA 1:200, and anti-MMP9 1:200.

Antigen retrieval was performed using CC1 antigen retrieval buffer (EDTA based) for collagen I, CC2 (citrate-based) antigen retrieval buffer for a-SMA and MMP9 and protease 2 enzyme-based pre-treatment for collagen IV. All the retrievals and immunohistochemistry staining were done using the Ventana Benchmark LT system (Ventana Medical Systems, Tucson, AZ, USA).

### Immunohistochemical (IHC) analysis

The samples were analyzed using light microscopy and sample pictures for each score were used to make the assessments more objective. To avoid subjectivity, two of the authors (HB & GB) performed the analysis and scoring of the individual sample separately. The results were then compared, and the mean value was calculated. The agreement of evaluation was measured by Cohen’s Kappa interrater reliability test, resulting in a mean coefficient of 0.86, indicating very good agreement.

For patients with multiple tumors (multifocal tumors), the largest one was chosen for analysis. Most of the patients in the pT1N1b group had several metastatic lymph nodes, and in these cases, we chose to stain and analyze several of these, when possible. The non-metastatic lymph nodes from the T1N0 group were not analyzed. Some cases contained both metastatic and non-metastatic lymph nodes: in these cases, the non-metastatic ones were used for comparison. When more than one metastatic lymph node was found on the same glass, the analysis was performed in such a way that each glass obtained a collected score, meaning that the expression in each lymph node contributed to the assessment and an average of these resulted in a score for the sample.

When looking at the PTC tissue samples, we evaluated trademarks for cancer such as papillary architecture, disorganization, large and clear nucleoli, mitoses, and the so-called “Orphan Annie-eyes,” that is, large round cells with a dense nucleus and clear cytoplasm, compared to the normal tissue.

The staining was evaluated based on *location*,* intensity*, and *distribution*.

**Fig. 1:**
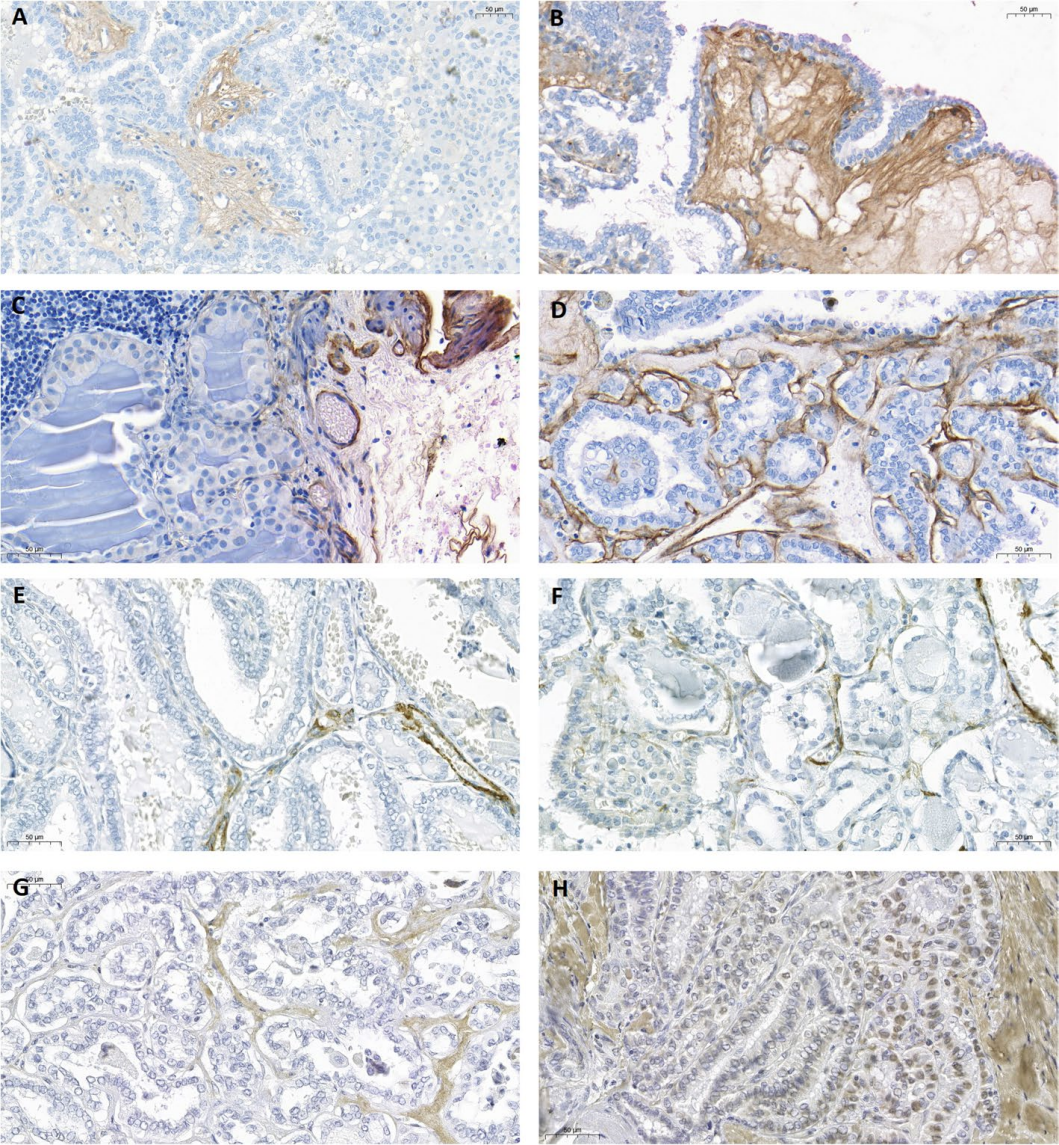
Immunohistochemical examples of various biomarkers and scores. **A** shows Col1 in PTC with slight expression and intensity in the tumor stroma. Normal location. **B** shows Col1 in PTC with strong expression and intensity in the tumor stroma. Normal location. **C** shows Col4 in a lymph node with no expression in the metastatic tumorous tissue. Normal expression and location in the normal lymphatic tissue nearby. **D** shows Col4 in PTC with strong distribution and intensity in the basement membrane and tumor stroma. Abnormal expression and location of the collagen type IV fibers. **E** shows a-SMA in PTC with slight distribution and intensity. Normal location. **F** shows a-SMA in PTC with medium distribution and intensity. Abnormal location. **G** shows MMP-9 in PTC with slight distribution and intensity. Normal location. **H** shows MMP-9 in PTC with strong distribution and intensity. Abnormal location. Pictures were taken using the Panoramic P250 FIII slide scanner (3DHISTECH) and reproduced using CaseViewer (3DHISTECH) at a 50-μm scale

*The location* of the different stains had to be analyzed regarding staining in unexpected patterns, since it is expected with some expression of Col1, Col4, a-SMA, and MMP-9 in both types of samples, even if normal tissue. The scoring was based on how much the staining’s expression in the metastatic areas differed from the non-metastatic parts of the lymph node (normal part) and was performed in the same way for tumorous vs non-tumorous areas in the thyroid samples. Details of the location scoring system are found in Table 1 and Fig. 1*.*

**Table 1:** Scoring system for Col1, Col4, a-SMA, and MMP-9—location score

Score	Expression of Col1	Expression of Col4	Expression of a-SMA	Expression of MMP-9
0	No expression	No expression	No expression	No expression
1	Same expression as in the healthy parts of the thyroid/lymph node sample. Col1-expression in connective tissue, fibrous capsule, and trabeculae	Same expression as in the healthy parts of the thyroid/lymph node sample. Col4 expression in the basement membrane (BM)	Same expression as in the healthy parts of the thyroid/lymph node sample. a-SMA expression in the vessels and myofibroblasts	Same expression as in the healthy parts of the thyroid/lymph node sample. MMP-9 expression in the epithelial cells
2	Different expression in the tumor stroma and in-between cancerous cells compared with the healthy tissue, such as differences in fiber thickness, intracytoplasmic/nuclear expression, and loss of structure	Different expression in the tumor stroma and in-between cancerous cells compared with the healthy tissue, such as loss of structure (BM) and fiber thickness	Different expression in the tumor stroma and in-between cancerous cells compared with the healthy tissue, such as differences in fiber thickness, myofibroblast differentiation	Different expression in the tumor stroma and in-between cancerous cells compared with the healthy tissue, such as intracytoplasmic/nuclear expression

*The intensity* of the positive-stained areas in the tumorous areas or metastatic lymph nodes was scored as follows: 0, none; 1, weak; 2, moderate; and 3, strong.

*The distribution* of the staining was assessed with scores from 0 to 4, purely based on how much percentage of the tumor’s stroma, or metastatic area of the sample, had a positive stain, as shown in Table 2*.* A score of 0 means that there was a completely negative stain, i.e., a negative control. A score from 1 to 4 was then given when there was a positive stain surrounding the cancerous or follicular cells, with a focus on the fibers surrounding/between rows of cells. In the case of MMP-9, we even looked at the distribution of the tumor epithelial cells. When analyzing the lymph nodes, the capsule (in the case of Col1) and the vessels (in the case of Col4 and a-SMA) were disregarded, since these are expected to have a positive stain in all samples. We also applied the distribution score for the healthy parts of our specimens in order to compare it with the pathological one. Details of the distribution scoring system are found in Table 2 and Fig. 1.

**Table 2:** Scoring system for Col1, Col4, a-SMA, and MMP-9—distribution score (percentage) in the tissue

Score	Expression (%) of Col1/Col4/a-SMA/MMP-9
0	Negative stain
1	1–25% staining
2	26–50% staining
3	51–75% staining
4	76–100% staining

When scoring samples with multiple lymph nodes in the same sample, the metastatic parts of each were assessed to a combined score for the sample. In some of the thyroid samples, thyroiditis and/or goiter were present, and in these cases, the healthiest areas were used for comparison with the tumor.

Both the intensity and the distribution scores were added together to create *a total score* (0–7 points).

Cutoff levels for the percentage of cells with a positive stain within the metastatic/cancerous stroma/between healthy follicular cells.

### Statistical methods

SPSS (Version 25.0, SPPS Inc., Chicago, IL, USA) was used for the statistical analysis. Fischer’s exact test was used for gender comparison. Mann–Whitney *U* test was used for the mean comparison between the N0T and N1T/N1N groups. The Wilcoxon signed-rank test was used for median comparison between the metastatic tumors (N1T) and their corresponding lymph nodes (N1N). The Spearman’s rho nonparametric test was used to check for correlations between the metastatic tumors (N1T) and their corresponding lymph nodes (N1N). We calculated the Cohen coefficient *κ* for the statistical analysis of interobserver reliability. A value of 1 means total agreement, and a value of 0 means that the agreement does not exceed the random measure. The graphs were created using GraphPad Prism (Version 9, GraphPad Software, San Diego, CA, USA).

## Results

### Patient characteristics

A comparison of the two groups, divided by nodal status, is summarized in Table 3. The average age at diagnosis differed only slightly between the two groups. The mean tumor size in mm did not differ between the groups, and tumor size range was equal. The female/male ratio was higher in both groups. No significant differences were found between males and females when comparing the mean values of the distribution and total scores of the different stromal biomarkers regarding the different tumor groups (N0 and N1) as shown in Supplementary Table 1. No significant correlation was found between age and the mean value of the distribution and total scores of the different stromal biomarkers in both tumor groups (N0 and N1).

**Table 3:** Patient characteristics, divided by nodal status

		pT1N0 non-metastatic tumors (***N*** = 25)	pT1N1b metastatic tumors (***N*** = 19)
Mean average age at diagnosis years (range)		58.9 (26–84)	53.7 (28–79)
Gender (female/male)		23/2	12/7
Mean tumor size mm (range)		11.4 (1.6–20)	10.3 (1.3–20)
Growth pattern	Purely papillary	23/25	19/19
	Follicular variant	2/25	0/19
Multifocality		5/25	3/19
Average number of metastastic lymph nodes^a^		-	9 (1–26)

^a^The average number of lymph nodes has been calculated without regard to central or lateral location

### Expression of Col1

**Fig. 2:**
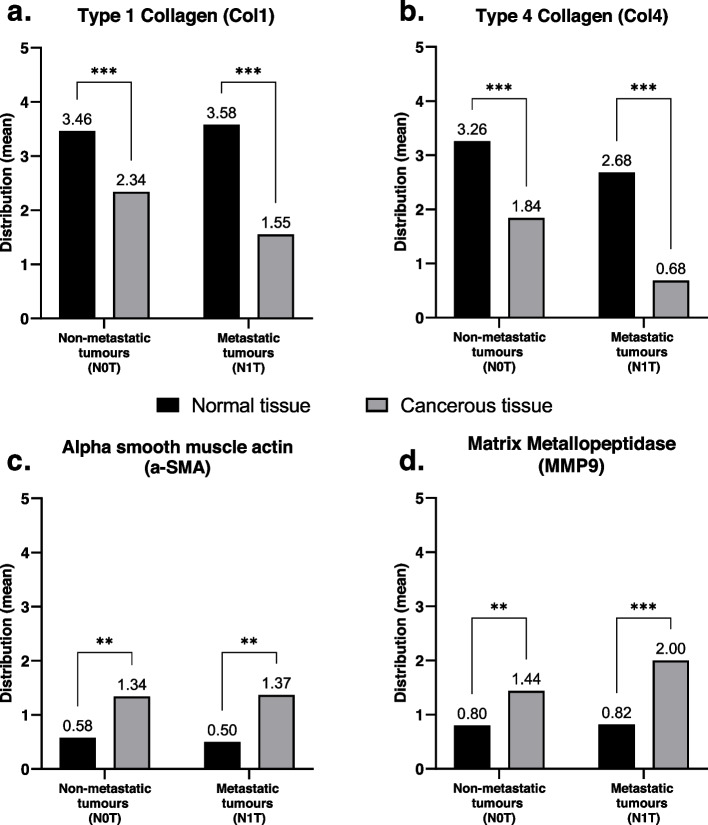
Comparison of the mean distribution scores (*Y*-axis) in the normal and tumorous tissues divided by the tumor subgroups, non-metastatic (N0T) vs. metastatic (N1T) (*X*-axis). Col1 (**a**) and Col4 (**b**) are significantly higher in the normal tissue. a-SMA (**c**) and MMP9 (**d**) are significantly higher in the cancer tissue. Black bars represent the normal tissue. Gray bars represent the tumorous tissue. **p <0.01, ***p <0.001

Col1 was largely expressed between follicles in healthy samples. It was mostly found as dense fibers, but in some parts of the samples, there was also a more granulated or clustered expression (Fig. 1a, b). If the PTC was encapsulated, Col1 was expressed in great amounts in the entire capsule. Col1 was expressed significantly more in the normal tissue compared with PTC samples (*p* < 0.001) (Fig. 2a). There were no differences in location score between normal thyroid tissue and cancer tissue when comparing the tumors in the N0 vs. N1 groups (Table 4), indicating a similar expression pattern in all groups, including the normal tissue.

**Table 4:** Mean location, distribution, and total scores in Col1, Col4, a-SMA, and MMP-9

		*Col1*	*Col4*	*a-SMA*	*MMP9*
*Location score*	Non-metastatic tumors (N0T)	1.05	1.40*	1.74*	1.52*
	Metastatic tumors (TN1T)	1.10	1.37*	1.79*	1.55*
	Metastatic lymph nodes (N1N)	1.10	1.58*	1.79*	1.63*
*Distribution score*	Non-metastatic tumors (N0T)	2.38*	1.84*	1.34	1.44
	Metastatic tumors (N1T)	1.55	0.68	1.37	2.00
	Metastatic lymph nodes (N1N)	1.66	0.71	1.82	1.92
*Total score*	Non-metastatic tumors (N0T)	5.00*	4.32**	3.18	2.88
	Metastatic tumors (N1T)	4.13 +	2.74	3.21	3.53 +
	Metastatic lymph nodes (N1N)	4.32 +	2.60	3.82	3.45 +

^*^Significant differences *p* < 0.05, **Significant differences *p* < 0.01

+ Positive correlation between N1T and N1N using Spearman rho analysis (0.05 level significance)

**Fig. 3:**
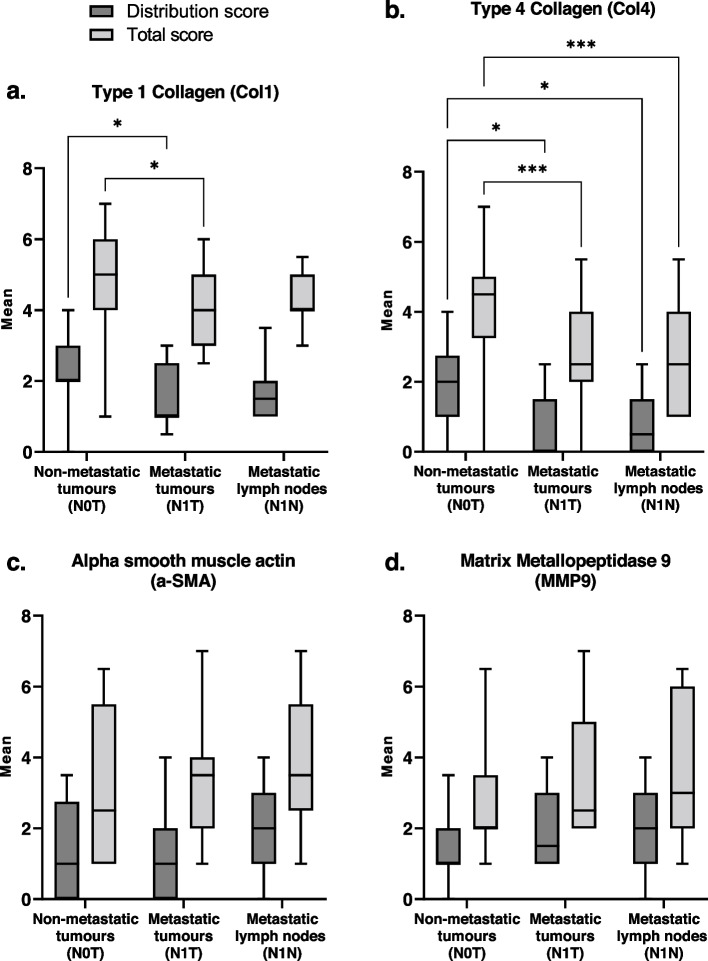
Comparison of the mean distribution (dark gray bars) and total scores (light gray bars) (*Y*-axis) divided by the different tumor groups (N0T vs. N1T) and the metastatic lymph nodes (N1N) (*X*-axis). Col1 (**a**) is significantly higher in the non-metastatic tumors (N0T) compared with the metastatic ones (N1T). Col4 (**b**) is significantly higher in the non-metastatic tumors (N0T) compared with the metastatic ones (N1T) and even with the metastatic lymph nodes (N1N). *p <0.05, ***p <0.001

As seen in Table 4, both the means of distribution and total scores were significantly higher in the N0 group tumors (N0T) (*p* < 0.05) compared with the tumors in the N1 group (N1T) (Fig. 3a). There was no difference in distribution and total scores between the tumors and their respective lymph nodes (N1T vs N1N). However, a positive significant correlation was expressed (0.533 + , *p* < 0.05 for the distribution score and 0.700 + , *p* < 0.001 for the total score). This indicates that expression of the Col1 in both the tumors and their respective lymph nodes was similar.

### Expression of Col4

Col4 occurs naturally in basement membranes and was seen in the samples as delicate brown fibers between follicles (Fig. 1c, d). In some samples, there was a fine granulated expression together with fine fibers, as well as dense fibers around the blood vessels in both normal tissue and the tumors. Col4 was expressed more significantly in the normal tissue compared with the cancer tissue (*p* < 0.001) (Fig. 2b). The location score was significantly higher in the cancer tissue and metastatic lymph nodes compared with the healthy tissue (Table 4), indicating loss of basement membrane in the stromal reaction. No difference was observed when comparing different tumor groups, N0T vs. N1T, indicating that Col4 was expressed equally abnormally in both tumor groups as well as in the metastasized lymph nodes N1N (Table 4).

As seen in Table 4, both the means of distribution and total scores were significantly higher in the N0 group tumors (N0T) (*p* < 0.05 and *p* < 0.001, respectively) compared with the tumors in the N1 group (N1T) (Fig. 3b). There was no difference in the distribution and total scores between the tumors and their respective lymph nodes (N1T vs N1N), and a slight positive but non-significant correlation could be seen.

### Expression of a-SMA

a-SMA is expressed in the vascular smooth muscle cells and myofibroblasts. Myofibroblast proliferation was noted, in the tumor stroma, and myofibroblast to fibroblast transformation also occurred. The intensity and distribution tended to increase near the capsule in the encapsulated tumors. The a-SMA was expressed more significantly in the tumors compared with the normal tissue (*p* < 0.001) *(Fig. *2c*).* The location score was significantly higher in the cancer tissue and metastatic lymph nodes compared with healthy tissue (Table 4), indicating abnormal histopathological expression in the cancer tissue such as fiber thickness and myofibroblast differentiation. No difference was observed when comparing the different tumor groups, N0T vs. N1T, indicating that the a-SMA was equally abnormally expressed in both tumor groups as well as in the metastasized lymph nodes, N1N (Table 4).

As seen in Table 4, both the means of distribution and total scores did not differ significantly between the tumors in the N0 group (N0T) compared with those in the N1 group (N1T) (Fig. 3c). The means of the distribution and total scores trended slightly higher in the metastasized lymph nodes compared to their primary tumors (N1T vs N1N), and a slight negative but non-significant correlation could be seen.

### Expression of MMP-9

MMP-9 is normally expressed intracellularly in the epithelial cells. During the stromal reaction and interaction, it is expected to be expressed in the extracellular matrix and tumor stroma. Here as well, the intensity and distribution tended to increase near the capsule in the encapsulated tumors. MMP-9 was expressed more significantly in the tumors compared with the normal tissue (*p* < 0.001) (Fig. 2d). The location score was significantly higher in the cancer tissue and metastatic lymph nodes compared with the healthy tissue (Table 4), indicating abnormal histopathological expression in the cancer tissue such as intracytoplasmic/intranuclear localization of the MMP-9. No difference was observed when comparing different tumor groups, N0T vs. N1T, indicating that the MMP-9 was equally abnormally expressed in both tumor groups and in the metastasized lymph nodes N1N (Table 4).

As seen in Table 4, both the means of distribution and total scores did not differ significantly between the tumors in the N0 group (N0T) compared with those in the N1 group (N1T), being slightly higher in the N1T tumor group (Fig. 3d). There was no difference in distribution and total scores between the tumors and their respective lymph nodes (N1T vs N1N). However, a positive significant correlation was expressed (0.490 + , *p* < 0.05 for the distribution score and 0.530 + , *p* < 0.05 for the total score). This indicates that the expression of the MMP-9 in both the tumors and their respective lymph nodes was similar and slightly higher than in the N0T group.

The location score was compared with that of the healthy tissue (which has a score of 1) and even between the subgroups.

## Discussion

Based on the location score, the results indicated that the expression of Col4, a-SMA, and MMP-9 differed histopathologically between normal and cancerous thyroid tissue. The location score showed a significant difference between these biomarkers and normal tissue regarding the morphology of the fibers and the abnormal location expression. This indicates that the stromal reaction in the PTC and the metastatic lymph nodes in general alters the expression of these biomarkers, as an indicator and predictor for aggressiveness and invasiveness.

Interestingly, the results show that the distribution scores in both Col1 and Col4 were significantly lower in the tumors than in the normal tissue (Fig. 2a, b). The same goes for the distribution and total scores in the non-metastasized tumor group (N0T), which were significantly higher than the tumors in the metastasized group (N1T) (Fig. 3a, b).

These findings could point toward a loss of Col1 in tumors, especially the metastasized ones, which was reinforced by the fact that there was a correlation between the tumors (N1T) and their corresponding lymph nodes. As shown in previous studies, Col1 has been found to affect cell adhesion, migration, and proliferation. In other words, Col1 has some effect on cancer progression and, in specific, may play a protective role against metastasis as collagens break down due to collagenases and matrix metalloproteases in the tumor stroma in aggressive tumors [26, 31]. These patterns of expression seem logical in the case of Col4 which normally stains the BM. It has been shown that the BM breaks down as the tumor grows more invasively [27, 28], promoting metastasis and aggressiveness. The expression of Col4 in the tumors (N1T) and their corresponding lymph nodes (N1N) had a moderate, but non-statistically significant correlation, which, as in the case with Col1, reinforces the positive finding of Col4 loss in the cancer tissue in general and in the metastasized tissue in specific.

The analysis of the a-SMA showed a significant difference in expression between the cancerous and the normal tissues, with higher distribution means in the tumors (Fig. 2c). When comparing the non-metastasized tumor group (N0T) with the metastasized tumor group (N1T), the analysis showed a trend towards a higher expression of the distribution and total scores. There was no significant correlation between the primary tumors (N1T) and their corresponding lymph nodes (N1N). These findings might indicate tendencies to increased expression in the metastasized tumor group which usually has a worse prognostic outcome. This has been shown in several other studies with other cancer forms but, to our knowledge, not yet in thyroid cancer. The a-SMA is expressed in the myofibroblasts which appear to be increased in the tumor stroma as part of the myofibroblastic proliferation, which at some point, can lead to fibroblast transformation and loss of myofibrils. This might explain why expression was not significantly higher in the metastasized group, in which the stromal process and desmoplastic reaction are expected to be more aggressive.

MMP-9 analysis produced results similar to those for a-SMA. There were significant differences in expression between the cancerous and normal tissues, with higher distribution means in the tumors (Fig. 2d). This agrees with results from other studies. Distribution was higher but not significant in the metastasized tumor group (N1T) compared with the non-metastasized (N0T), and there was a positive correlation between the metastasized tumors (N1T) and their corresponding lymph nodes (N1N). Some studies have shown that higher MMP-9 levels correlate with a worse prognosis for patients with PTC and more persistent and recurrent metastasized disease. As a protease, MMP-9 influences the tumor stromal remodeling and might be involved in the breakdown of the basement membrane. This could explain the inverted results in the expression of Col1 and Col4 versus MMP-9.

As mentioned earlier, the BRAF V600E mutation is the most common genetic alteration found in thyroid cancer, particularly PTC, and is associated with more aggressive disease. A previous study from Jolly et al. [13] has shown increased levels of Col1 in the tumor stroma of BRAF V600E-positive thyroid cancer patients with loss of PTEN. Recruitment, proliferation, and activation of tumor-associated fibroblasts in the tumor microenvironment leads to more collagen fibril deposition and crosslinking, especially in the poorly differentiated tumors [13]. These findings partially contradict ours. However, we did not perform BRAF analysis on our specimens and the specimens were not routinely checked for BRAF mutation during the time the primary specimens were collected. Further analysis of the BRAF mutations and the relationship with tumor stromal biomarkers would indeed be valuable. Also, the relationship with telomerase reverse transcriptase (TERT) promotor mutations has been shown to have an association with aggressive characteristics, recurrence, and mortality in PTC patients, especially when combined with BRAF V600E mutation, giving a synergistic impact [43, 44].

One of the strengths of this study is that the scoring was as objective as possible and done by two independent observers with a standardized scoring scale. Several strategies were used to make it even more objective, such as using exemplifying pictures and discussing uncertain scores and outliers. Still, the location scoring was difficult since there were no apparent patterns that could result in a more defined scoring system. A more sophisticated system for scoring, for all variables, or an AI-based algorithm for scoring, could result in much more refined results.

## Conclusions

The expression of the tumor biomarkers Col1, Col4, a-SMA, and MMP-9 in this cohort of small PTCs differed significantly from normal thyroid tissue. The expression of Col1 and Col4 in cancerous tissue was significantly lower compared to the healthy tissue and significantly higher in the non-metastasized tumors compared to the metastasized ones and their respective lymph nodes—indicating a possible protective effect. The opposite relationship was observed in the tumor biomarkers a-SMA and MMP-9 even though it was not statistically significant. These findings coincide with previous studies on biomarker expression in other cancer forms and very few do on PTC. Since the tumor microenvironment is multi-factorial and complex, an investigation of how several biomarkers interact is essential for a better understanding of the mechanisms behind tumorigenesis and will be a topic for further studies. The biomarkers investigated in this study can be considered interesting as potential markers for aggressiveness and invasiveness in PTC and, when combined with genetic analyses of, for example, BRAF V600E, and TERT promoter mutations, can potentially predict the progression and prognosis of PTC in order to favorably tailor the diagnosis, treatment, and follow-up of patients with PTC. However, this is a relatively small cohort, further and larger studies are required.

## Supplementary Information

**Additional file 1: Supplementary Table 1.** Gender comparison of the mean values of the distribution and total scores of the different stromal biomarkers (Col1, Col4, a-SMA and MMP-9) regarding the different tumour groups (N0T and N1T). No significant differences were found between males and females regarding the expression of stromal biomarkers.

## Data Availability

The datasets supporting the conclusions of this article are included within the article (and its supplemental table).

## Abbreviations

PTC: Papillary thyroid carcinoma
Col1: Type 1 collagen
Col4: Type 4 collagen
a-SMA: Alpha-smooth muscle actin
MMP9: Matrix metallopeptidase 9
FNA: Fine-needle aspiration
PTMC: Papillary thyroid microcarcinomas
MAPK: Mitogen-activated protein kinase pathway
ECM: Extracellular matrix
BM: Basement membrane
TERT: Telomerase reverse transcriptase
