# Supplementary material for: The expression of stromal biomarkers in small papillary thyroid carcinomas
Source: World J Surg Oncol. 2022 Oct 14;20:340. doi: 10.1186/s12957-022-02805-w (PMC9563774; doi:10.1186/s12957-022-02805-w)
Supplement: Supplementary file 1 — Additional file 1: Supplementary Table 1. Gender comparison of the mean values of the distribution and total scores of the different stromal biomarkers (Col1, Col4, a-SMA and MMP-9) regarding the different tumour groups (N0T and N1T). No significant differences were found between males and females regarding the expression of stromal biomarkers. [file 12957_2022_2805_MOESM1_ESM.docx]

# Supplementary data

*Supplementary Table 1:* Gender comparison of the mean values of the distribution and total scores of the different stromal biomarkers (Col1, Col4, a-SMA and MMP-9) regarding the different tumour groups (N0T and N1T). No significant differences were found between males and females regarding the expression of stromal biomarkers.

|  |  | | *Col1* | *Col4* | *a-SMA* | *MMP-9* |
| --- | --- | --- | --- | --- | --- | --- |
| Distribution score  (mean) | N0T | Males n=2 | 2.00 | 1.00 | 2.25 | 1.00 |
|  |  | Females n=23 | 2.37 | 1.91 | 1.26 | 1.48 |
|  |  | *p-value* | 0.644 | 0.285 | 0.176 | 0.414 |
|  | N1T | Males n=7 | 1.85 | 1.14 | 2.00 | 2.28 |
|  |  | Females n=12 | 1.37 | 0.41 | 1.08 | 1.83 |
|  |  | *p-value* | 0.203 | 0.158 | 0.121 | 0.502 |
| Total score  (mean) | N0T | Males n=2 | 5.00 | 2.50 | 5.25 | 2.50 |
|  |  | Females n=23 | 4.95 | 4.47 | 3.00 | 2.91 |
|  |  | *p-value* | 0.960 | 0.479 | 0.139 | 0.873 |
|  | N1T | Males n=7 | 4.28 | 3.07 | 4.00 | 4.07 |
|  |  | Females n=12 | 4.04 | 2.54 | 2.75 | 3.2 |
|  |  | *p-value* | 0.661 | 0.488 | 0.135 | 0.474 |
